# Evaluation of endothelial glycocalyx injury biomarkers in feline hemotropic mycoplasmosis

**DOI:** 10.1038/s41598-024-62359-7

**Published:** 2024-06-05

**Authors:** Merve Ider, Ceylan Ceylan, Amir Naseri, Onur Ceylan, Murat Kaan Durgut, Mahmut Ok, Suleyman Serhat Iyigun, Busra Burcu Erol, Hatice Betul Sahin, Merve Cansu Kilickaya

**Affiliations:** 1 Department of Internal Medicine, Faculty of Veterinary Medicine, Selcuk University, Konya, Turkey; 2 Department of Parasitology, Faculty of Veterinary Medicine, Siirt University, Siirt, Turkey; 3 Department of Parasitology, Faculty of Veterinary Medicine, Selcuk University, Konya, Turkey

**Keywords:** Biomarker, Cat, Endothelin-1, Glycocalyx, *Mycoplasma haemofelis*, Syndecan-1, Infectious-disease diagnostics, Anaemia

## Abstract

The present study aimed to investigate endothelial glycocalyx (eGCx) damage in cats with feline hemotropic mycoplasmosis caused by *Mycoplasma haemofelis* using selected biomarkers and to determine the diagnostic and prognostic significance of these biomarkers. The study included 25 cats with feline hemotropic mycoplasmosis and 10 healthy cats. Clinical examination, blood gas analysis, complete blood count, and biochemical analysis were performed. Hemotropic mycoplasmosis diagnosed by microscopic examination and molecularly confirmed by PCR targeting the *Mycoplasma haemofelis 16s rRNA* gene. To evaluate endothelial glycocalyx damage, syndecan-1, endothelin-1 (ET-1), asymmetric dimethylarginine (ADMA), and vascular endothelial growth factor-A (VEGF-A) concentrations were measured using cat-specific commercial ELISA kits. Of the cats with feline hemotropic mycoplasmosis, 14 (56%) survived and 11 (44%) died. While syndecan-1 and ET-1 concentrations were significantly higher in cats with hemotropic mycoplasmosis compared to the control group (*p* < 0.001), no statistically significant difference was found for ADMA and VEGF-A concentrations (*p* > 0.05). Endothelial glycocalyx biomarkers showed significant correlations with each other and with hematological parameters (*p* < 0.01). The results of the ROC analysis showed that ET-1 with area under the curve (AUC) of 0.821 (*p* < 0.01) and VEGF-A with AUC of 0.805 (*p* < 0.010) were found to be significant prognostic indicators. In conclusion, this study demonstrated that serum syndecan-1 and ET-1 can be used as diagnostic and serum ET-1 and VEGF-A as prognostic biomarkers in cats with hemotropic mycoplasmosis. Our results indicate the development of eGCx damage in feline hemotropic mycoplasmosis and suggest that glycocalyx disruption may contribute to the pathogenesis of the disease.

## Introduction

The significance of vector-borne diseases has recently become more recognized due to ecological and climatic changes. Among these diseases, infections caused by hemotropic mycoplasma species play an essential role. Hemotropic mycoplasmas (hemoplasmas) are uncultivable bacteria that epierythrocytically infect red blood cells and can cause anemia by inducing hemolysis. *Mycoplasma haemofelis* is one of the most pathogenic feline hemoplasmas and can cause significant hemolytic disease even in immunocompetent cats. Although *Mycoplasma haemofelis* is less common in cats, it is reported to be responsible for acute clinical infections compared to other feline hemotropic *Mycoplasma* species^1,2^.

Endothelial glycocalyx (eGCx) is a 1–3 µm thick dynamic layer that covers the apical surface of all endothelial cells and maintains vascular hemostasis, tonus, and permeability^3,4^. This layer regulates leukocyte adhesion/migration by maintaining endothelial oncotic pressure and plays a vital role in inhibiting intravascular thrombosis^4,5^. It also controls vascular resistance through proper endothelial nitric oxide (NO) production and vasodilation^6^. Endothelial glycocalyx, which has attracted more attention in recent years, consists of glycoproteins containing sialic acid, proteoglycans (syndecan, glypican) which are membrane-bound core proteins, glycosaminoglycan chains (heparan sulfate, chondroitin sulfate) and long-chain hyaluronic acid (HA)^7^. When the glycocalyx layer is damaged, glycocalyx components are shed into the bloodstream and can be measured as biomarkers of glycocalyx breakdown. Among these biomarkers, syndecan proteoglycans, which are the basic structural component of the eGCx, play an important role. It has been stated that increased serum syndecan-1 concentrations are the main indicator of eGCx damage in rats and human^8,9^. It has been mentioned that eGCx shedding is also mediated by glycocalyx sheddases, such as heparanase, which are activated by reactive oxygen species and proinflammatory cytokines^10^. Endothelin-1 (ET-1) and asymmetric dimethylarginine (ADMA) are heparanase activators responsible for the breakdown of heparan sulfate (HS), which constitutes 50% to 90% of the glycosaminoglycan (GAG) family expressed on the surface of endothelial cells^7^. On the other hand, vascular endothelial growth factor-A (VEGF-A) controls vascular permeability, proliferation, survival, and inflammation of endothelial cells^11,12^. Vascular endothelial growth factor-A concentrations have been shown to be elevated in inflammatory diseases that may be the result of endothelial dysfunction in human^13,14^.

Various diseases such as sepsis^14^, malaria^9,15^, cardiovascular disease^16^ and COVID-19^17^ in humans and dogs with sepsis^18^ have been reported to cause eGCx damage^19^. Moreover, eGCx damage is closely related to the pathophysiology of these diseases^4^. This study was designed with the hypothesis that glycocalyx damage may also develop in cats with hemotropic mycoplasmosis. The primary aim of this study was to evaluate eGCx damage in feline hemotropic mycoplasmosis using glycocalyx damage biomarkers (syndecan-1, ET-1, ADMA, and VEGF-A) and to determine the diagnostic and prognostic significance of these biomarkers. Second, the relationship between biomarkers of glycocalyx damage and hematological/biochemical parameters was determined.

## Methods

### Ethical statement

The study protocol was approved by the Institutional Ethics Committee of the Faculty of Veterinary Medicine, Selcuk University (No. 2022/46), and conducted from February 2022 to September 2023. All methods were carried out in accordance with relevant guidelines and regulations and in compliance with the Animal Research: Reporting of In Vivo Experiments (ARRIVE) guidelines.

### Cat with hemotropic mycoplasmosis

Twenty-five owned cats of different breeds, ages, and sexes diagnosed with hemotropic mycoplasmosis and exhibiting clinical signs such as anemia, icterus, and depression were enrolled. All cats were owned and except 3 of them had access to the outdoor. The materials to be used in the microscopical, serological, and molecular analysis were collected from cats admitted to the Animal Hospital of the Faculty of Veterinary Medicine, Selcuk University for diagnosis and treatment. The study has not included cats with any disease other than hemotropic mycoplasmosis.

### Healthy cats

Ten healthy cats of different breeds, age and sex that were admitted to the Animal Hospital of the Faculty of Veterinary Medicine, Selcuk University for vaccination, preventive antiparasitic drug administration, and general control were enrolled. All the cats were owned and lived indoors. Healthy cats, which constituted the control group, were defined as the absence of hemotropic mycoplasmosis in microscopy and PCR analysis, rectal temperature within normal limits, and no use of antimicrobial or other drugs within 2 weeks before sampling. Healthy cats also had normal CBC, blood gas analysis and biochemistry.

### Collection of blood samples

Blood samples were collected from both healthy and hemotropic mycoplasmosis cats at the time of admission. Blood samples for blood gas analysis, complete blood count (CBC), biochemical and endothelial damage marker analyses were collected from the *vena cephalica antebrachium*. Plastic syringes containing sodium heparin were used for blood gas measurements. Tubes containing K_3_EDTA were used for CBC analysis. Blood gas and CBC measurements were performed within 5–10 min after sample collection. Non-anticoagulant tubes were used for serum collection. Blood samples collected for biochemical, and biomarker analyzes were kept at room temperature for 15 min and then centrifuged at 20 × *g* for 10 min. The sera were divided into two parts and one part was used for biochemical analysis and the other part was stored at -80 °C for biomarker analysis.

### Blood gas analysis

Venous blood pH, partial pressure of carbon dioxide (pCO_2_), partial pressure of oxygen (pO_2_), oxygen saturation (SO_2_), base deficit (BE), and bicarbonate (HCO_3_) were measured using an automated blood gas analyzer (ABL 90 Flex, Radiometer, Brea, CA, United States).

### Complete blood count (CBC) analysis

Total leukocyte count (WBC), lymphocyte (Lym), monocyte (Mon), granulocyte (Gra), erythrocyte count (RBC), mean corpuscular volume (MCV), mean corpuscular hemoglobin concentration (MCHC), red cell distribution width (RDW), hematocrit (HCT), hemoglobin (Hb) and platelet (PLT) levels were measured using an automated cell counter (MS4e, Melet Schlosing Laboratories, Osny, France).

### Biochemical analysis

Potassium (K), sodium (Na), calcium (Ca), chlorine (Cl), glucose (Glu), lactate (Lac) concentrations were measured using an automated blood gas analyzer (ABL 90 Flex, Radiometer, Brea, CA, United States). Blood urea nitrogen (BUN), creatinine (Cre), total bilirubin (Tbil), direct bilirubin (Dbil), alanine transaminase (ALT), aspartate aminotransferase (AST), alkaline phosphatase (ALP), lactate dehydrogenase (LDH), creatinine phosphokinase (CPK), gamma-glutamyl transferase (GGT), amylase (Amy), cholesterol (Chol), triglyceride (Tri), phosphorus (P), magnesium (Mg), total protein (Tpro), albumin (Alb) and albumin globulin ratio (A:G) values were measured by an autoanalyzer (Biotecnica BT 3000 Plus, Italy).

### Evaluation of endothelial glycocalyx biomarkers

Serum Syndecan-1 (Bioassay Technology Laboratory, Shanghai, China, Lot: E0068Cat), ET-1(Bioassay Technology Laboratory, Shanghai, China, Lot: E0095Cat), ADMA (Bioassay Technology Laboratory, Shanghai, China, Lot: E0092Cat), and VEGF-A (SunRed Biotechnology Co., Ltd, Shanghai, China, Lot: 201-28-1439) concentrations were measured using commercial feline-specific ELISA test kits according to the manufacturer’s instructions. The intra-assay coefficient of variation (CV), inter-assay CV, and minimum detectable concentrations (MDC) for biomarkers were < 8%, < 10%, and 0.025 ng/mL for Syndecan-1; < 8%, < 10%, and 1.18 ng/L for ET-1; < 8%, < 10% and 0.014 nmol/mL for ADMA; and < 10%, < 12% and 1.864 pg/mL for VEGF-A, respectively.

### Microscopic diagnosis of hemotropic mycoplasmosis

In this study, 0.5–1.5 mL blood samples were collected in anticoagulated tubes containing ethylenediamine tetraacetic acid (K_3_-EDTA) from 33 cats with suspected hemotropic mycoplasmosis based on clinical signs such as severe anemia, icterus and depression and hemogram findings. Thin blood smears were immediately prepared from all anticoagulated venous blood samples, dried in the open air, fixed with absolute methanol for 5 min, and stained with 10% Giemsa solution for 30 min. After staining, the thin blood smears were washed under tap water and dried. Immersion oil was dripped on the slides, and the smears were examined under 100× magnification of a light microscope (Leica DM1000) for epieritrocytically localized hemoplasmas. At least 50 microscopic fields were scanned to detect these haemopathogens.

### Molecular confirmation of hemotropic mycoplasmosis

The presence of the primary causative agent of feline hemotropic mycoplasmosis, *Mycoplasma haemofelis*, was also molecularly investigated in the blood samples of all sera to be used in enzyme-linked immunosorbent assays. Accordingly, genomic DNA was first extracted from blood samples using the High Pure PCR Template Preparation Kit (Roche, Germany) following the manufacturer's instructions. The extracted genomic DNA was stored at − 20 °C until molecular analysis. Each DNA sample was then screened by species-specific PCR targeted to amplify the *M. haemofelis 16S rRNA* gene fragment (170 bp). Molecular screening was performed according to the procedure previously described by Jensen et al.^20^. The final volume of the reaction mixture was 10 µL and consisted of 2 µL 5× One Taq Standard Reaction buffer (BioLabs, New England), 0.2 µL dNTP mix (Deoxynucleotide solution mix, BioLabs, New England), 0.2 µM each primer (Hf-F: 5′-ACG AAA GTC TGA TGG AGC AAT A-3′ and Hf-R: 5′-ACG CCC AAT AAA TCC GRA TAA T-3′), 0.05 µL Taq DNA polymerase (BioLabs, New England), 1 µL DNA template and 6.35 µL double distilled water (Invitrogen, UltrapureTM Distilled Water, DNAse/RNAse-Free). *Mycoplasma haemofelis* positive genomic DNA sample previously confirmed by sequence analysis (OR979169, https://www.ncbi.nlm.nih.gov/nuccore/OR979169) was used as positive control and double distilled water (Invitrogen, UltrapureTM Distilled Water, DNAse/RNAse-Free) was used as a negative control. PCR amplicons were electrophoresed on 1.5% agarose gels, stained with ethidium bromide, and visualized under a UV transilluminator (UVP, Upland, CA, USA). Considering the co-infection status in cats, all blood samples included in the study were molecularly confirmed to be negative for some other haemopathogens such as *Babesia sp*., *Rickettsia sp*., *Bartonella sp*. and *Anaplasma* *sp*. For this purpose, PCR analyses utilizing universal primers for the amplification of *Babesia/Theileria/Hepatozoon* sp. 18S rRNA, *Anaplasma/Ehrlichia* sp. 16S rRNA, *Rickettsia* sp. glt A and *Bartonella* sp. 16-23S gene fragments were performed following the conditions described in the relevant literature^20–23^. The primer pair (HF-F/R) used in this study for the molecular identification of *Mycoplasma haemofelis* is also used for the identification of *Candidatus Mycoplasma haemominutum* (CMh), another less pathogenic feline haemotropic *Mycoplasma* species. This primer can be used to amplify 170 bp fragments of the 16S rRNA gene for *Mycoplasma haemofelis* and 193 bp fragments of the same gene for CMh^20^. In addition, the molecular negativity of another feline haemoplasma species, *Candidatus Mycoplasma turicensis* was checked by PCR targeting the amplification of the 488 bp region of the 16S rRNA gene of this species^24^.

### Statistical analysis

The data of this study were analyzed using SPSS 25 (IBM Corp. Released 2017. IBM SPSS Statistics for Windows, Version 25.0 Armonk, NY: IBM Corp.) statistical program. One Sample Kolmogorov–Smirnov test was applied to evaluate the prerequisites for normal distribution (parametric or non-parametric) of the data. Since the study data showed non-parametric distribution, they were presented as median and interquartile range (IQR, 25th–75th percentile). For comparison between groups, the Mann–Whitney U test was used. The Spearman rank correlation test was used to determine the correlation between variables. In Spearman rank correlation analysis, the scale ranges from -1 to 1, with values close to 1 indicating a strong correlation and values close to 0 indicating a weaker correlation. In addition, Receiver Operating Characteristic (ROC) analysis was performed to determine the sensitivity, specificity, and cut-off values of selected variables in predicting mortality in surviving and nonsurviving cats. Statistical significance was considered as *p* < 0.05.

## Results

### Clinical findings

Cat breeds included in the hemotropic mycoplasmosis group (16 male, 9 female); 15 mix breed, 3 Turkish Angora, 2 Tuxedo, 1 Turkish Van, 1 Scottish Fold, 1 British Shorthair, 1 Siamese and 1 Persian. The cat breeds included in the control group (4 male, 6 female) were 6 mix breed, 2 Scottish Fold, 1 Turkish Angora, 1 Turkish Van. The median age was 3 (1–4) and 2 (1–4) years for healthy and cat with hemotropic mycoplasmosis, respectively (p > 0.05). In 25 M*. haemofelis-*positive cats, the most prominent clinical signs were lethargy (n:25), anorexia (n:25), and dehydration (n:15). Icterus (n:20), prolonged capillary refill time (n:24), pale yellow mucous membranes (n:21) were observed. Of the cats, 9 were hyperthermic (> 40 °C), 9 were normothermic and 7 were hypothermic (< 35 °C), respectively. Of the hemotropic mycoplasmosis cats, 11 (44%) did not survive and 14 (56%) survived.

### Microscopic examination findings

Microscopic examination of thin blood smears commonly revealed *M. haemofelis*-infected erythrocytes as well as some abnormalities including ghost RBCs, erythrocyte agglutination, macrocytosis, anisocytosis, reticulocytosis and Howell-Jolly bodies, with rare presence of spherocytes.

### Blood gas and CBC analysis

Venous blood gas and complete blood count parameters of healthy and infected cats are presented in Table 1. While BE and HCO_3_ levels of cats infected with *M. haemofelis* were significantly lower than healthy cats (*p* < 0.05). In CBC analysis, WBC, Lym, Mon, Gra, and RDW levels of cats with hemotropic mycoplasmosis were significantly higher, while RBC, Hct, Hb, and PLT levels were lower (*p* < 0.05).

**Table 1:** Venous blood gas and CBC parameters of healthy and infected cats.

Parameters	Healthy cats (n:10)	Cats with hemotropic mycoplasmosis (n:25)	*p* value
pH	7.37 (7.35 to 7.39)	7.33 (7.30 to 7.39)	0.52
pCO_2_ (mmHg)	30.05 (29.07 to 34.35)	29.20 (25.82 to 33.02)	0.30
pO_2_ (mmHg)	42 to 20 (36.60 to 44.85)	34.65 (26.05 to 45.70)	0.12
SO_2_ (%)	67.75 (53.87 to 72.55)	49.05 (33.02 to 69.52)	0.09
BE (mmol/L)	− 4.25 (− 4.63 to − 3.02)	− 9.75 (− 12.50 to − 7.57)	0.000
HCO_3_ (mmol/L)	19.45 (18.30 to 20.45)	16.65 (14.72 to 18.35)	0.001
WBC (cells/mL)	10.53 (8.20 to 11.06)	37.15 (24.02 to 49.57)	0.000
Lym (cells/mL)	3.38 (2.56 to 4.13)	8.35 (4.65 to 15.47)	0.004
Mon (cells/mL)	1.07 (0.72 to 1.65)	2.15 (0.84 to 2.49)	0.04
Gra (cells/mL)	5.68 (4.01 to 7.45)	22.31 (13.13 to 39.67)	0.000
RBC (× 10^3^ cells/mL)	9.25 (8.34 to 10.81)	3.90 (2.40 to 5.88)	0.000
MCV (fL)	48.80 (47.75 to 51.20)	51.85 (45.55 to 68.57)	0.21
MCHC (g/dL)	28.65 (23.92 to 32.55)	30.10 (23.22 to 32.97)	0.86
RDW	11.90 (11.42 to 12.57)	12.45 (11.92 to 11.87)	0.04
Hct (%)	46.70 (41.05 to 55.97)	18.65 (15.27 to 23.30)	0.000
Hb (g/dL)	11.80 (10.32 to 15.32)	4.75 (3.45 to 6.90)	0.000
PLT (cells/mL)	149.00 (132.00 to 183.75)	51.00 (30.25 to 81.00)	0.000

Data being expressed as median and interquartile range (IQR, 25th–75th percentile).

Power of Hydrogen (pH), Partial pressure of carbon dioxide (pCO_2_), partial pressure of oxygen (pO_2_), oxygen saturation (SO_2_), base deficit (BE), bicarbonate (HCO_3_), total leukocyte count (WBC), lymphocyte (Lym), monocyte (Mon), granulocyte (Gra), erythrocyte count (RBC), mean corpuscular volume (MCV), mean corpuscular hemoglobin concentration (MCHC), red cell distribution width (RDW), hematocrit (HCT), hemoglobin (Hb), platelets (PLT).

### Biochemical analysis

Biochemical parameters of healthy and cats with hemotropic mycoplasmosis are presented in Table 2. Lactate, TBil, DBil, AST, LDH, and P levels of cats with hemotropic mycoplasmosis were significantly higher than healthy cats, but albumin and A:G ratio were lower (*p* < 0.05). No statistically difference was detected in other biochemical parameters (*p* > 0.05).

**Table 2:** Biochemical analysis parameters of healthy and infected cats.

Parameters	Healthy cats (n:10)	Cats with hemotropic mycoplasmosis (n:25)	*p* value
K (mmol/L)	4.00 (3.47–4.25)	3.70 (3.57–4.32)	0.95
Na (mmol/L)	156.50 (155.75–160.00)	158.50 (154.00–163.25)	0.53
Ca (mmol/L)	1.05 (0.88–1.17)	0.96 (0.87–1.14)	0.61
Cl (mmol/L)	122.00 (118.75–124.25)	123.0 (120.25–126.50)	0.45
Glucose (mg/dL)	103.50 (90.75–117.25)	107.00 (89.75–137.50)	0.76
Lac (mmol/L)	1.55 (1.18–1.70)	2.60 (1.37–3.32)	0.01
BUN (mg/dL)	21.85 (19.47–27.52)	27.18 (13.95–36.56)	0.67
Cr (mg/dL)	1.20 (0.97–1.32)	0.90 (0.46–1.52)	0.33
Tbil (mg/dL)	0.50 (0.35–0.67)	4.55 (1.23–7.44)	0.000
Dbil (mg/dL)	0.20 (0.10–0.30)	1.09 (0.33–2.71)	0.000
ALT (U/L)	67.50 (49.50–72.00)	59.25 (41.24–127.36)	0.78
AST (U/L)	27.50 (21.50–55.00)	80.92 (41.00–134.00)	0.01
ALP (U/L)	24.00 (15.50–47.00)	25.02 (14.00–35.59)	0.78
LDH (U/L)	170.00 (117.50–264.50)	481.60 (265.59–878.84)	0.001
CPK (U/L)	130.50 (69.00–364.75)	287.70 (148.55–571.00)	0.11
GGT (U/L)	2.00 (1.00–3.25)	3.00 (1.71–4.00)	0.21
Amy (U/L)	1338.00 (823.00–1534.75)	1384.1 (966.86–1900.35)	0.55
Chol (mg/dL)	143.50 (112.75–151.75)	145.05 (98.72–184.00)	0.70
TG (mg/dL)	59.00 (42.00–74.00)	74.20 (49.35–175.35)	0.09
P (mg/dL)	5.05 (4.37–5.62)	6.25 (4.95–7.61)	0.02
Mg (mg/dL)	2.40 (2.07–2.55)	2.05 (1.53–2.54)	0.11
TP (g/dL)	6.95 (6.37–7.35)	7.74 (5.81–9.12)	0.13
Alb (g/dL)	3.20 (2.57–3.72)	2.73 (2.22–2.90)	0.02
A:G	0.98 (0.59–1.18)	0.52 (0.39–0.78)	0.01

Data being expressed as median and interquartile range (IQR, 25th–75th percentile).

Potassium (K), sodium (Na), calcium (Ca), chlorine (Cl), glucose (Glu), lactate (Lac), blood urea nitrogen (BUN), creatinine (Cr), total bilirubin (Tbil), direct bilirubin (Dbil), alanine transaminase (ALT), aspartate aminotransferase (AST), alkaline phosphatase (ALP), lactate dehydrogenase (LDH), creatinine phosphokinase (CPK), gamma-glutamyl transferase (GGT), amylase (Amy), cholesterol (Chol), triglyceride (TG), phosphorus (P), magnesium (Mg), total protein (TP), albumin (Alb), albumin globulin ratio (A:G).

### Biomarker analysis

Biomarker concentrations of healthy and cats with hemotropic mycoplasmosis are presented in Table 3. Syndecan-1 and ET-1 concentrations of cats with hemotropic mycoplasmosis were significantly higher than the healthy ones (*p* < 0.001). There was no significant difference in ADMA and VEGF-A concentrations between healthy and cats with hemotropic mycoplasmosis (*p* > 0.05) (Table 3).

**Table 3:** Biomarker concentrations in healthy and infected cats.

Parameters	Healthy cats (n:10)	Cats with hemotropic mycoplasmosis (n:25)	*p* value
Syndecan-1 (ng/mL)	2.09 (1.76–2.45)	3.14 (2.74–3.59)	0.000
ET-1 (ng/L)	28.28 (24.08–39.34)	93.36 (77.29–100.99)	0.000
ADMA (nmol/mL)	2.73 (2.25–2.19)	2.94 (2.35–3.14)	0.22
VEGF-A (pg/mL)	85.52 (43.56–101.88)	61.81 (51.13–90.88)	0.48

Data being expressed as median and interquartile range (IQR, 25th–75th percentile).

Syndecan-1, Endothelin-1 (ET-1), Asymmetric dimethylarginine (ADMA), Vascular endothelial growth factor-A (VEGF-A).

### Correlation analysis

Correlations between endothelial glycocalyx biomarker concentrations and some hematologic parameters determined in healthy and infected cats by Spearman correlation analysis are presented in Table 4. ET-1 concentration in blood serum showed a strong and positive correlation (*p* < *0.01*) with syndecan-1. ADMA showed a weak positive correlation with syndecan-1 concentrations (*p* < *0.05*), while ET-1 showed a moderate positive correlation (*p* < *0.01*). There was a strong positive correlation (*p* < *0.01*) between VEGF-A and ADMA concentrations. WBC levels showed a strong positive correlation (*p* < *0.01*) with syndecan-1 and a moderate positive correlation (*p* < *0.01*) with ET-1. RBC levels were strongly and negatively correlated with syndecan-1 (*p* < *0.01*) and ET-1 (*p* < *0.01*) concentrations. However, there was a moderate and negative correlation with WBC (*p* < *0.01*). There was a moderate and negative correlation between PLT levels and syndecan-1 (*p* < *0.01*) and ET-1 (*p* < *0.01*) concentrations, while there was a strong positive correlation with RBC (*p* < *0.01*). Among the biochemical parameters, lactate had a moderate positive correlation with ET-1 (*p* < *0.01*) and WBC (*p* < *0.01*), while A:G ratio had a moderate and negative correlation with VEGF-A (*p* < *0.01*).

**Table 4:** Correlations between biomarker concentrations and some hematologic parameters in healthy and infected cats.

Parameters	Syn-1	ET-1	ADMA	VEGF-A	WBC	RBC	PLT	Lac	A:G
Syn-1	1	0.67**	0.35*	0.07	0.65**	− 0.62**	− 0.56**	0.25	− 0.33
ET-1		1	0.48**	0.12	0.53**	− 0.62**	− 0.49**	0.55**	− 0.21
ADMA			1	0.65**	0.11	− 0.17	− 0.21	0.34	− 0.30
VEGF-A				1	− 0.06	0.10	0.01	− 0.01	− 0.55**
WBC					1	− 0.59**	− 0.41	0.45**	− 0.11
RBC						1	0.70**	− 0.34	0.26
PLT							1	− 0.25	0.27
Lac								1	− 0.23
A:G									1

Syndecan-1 (Syn-1), Endothelin-1 (ET-1), asymmetric dimethylarginine (ADMA), vascular endothelial growth factor-A (VEGF-A), Total leukocytes (WBC), erythrocytes (RBC), platelets (PLT), Lactate (Lac), albumin globulin ratio (A:G).

**Correlation is significant at the 0.01 level,

*Correlation is significant at the 0.05 level.

### Mortality analysis

**Figure 1:**
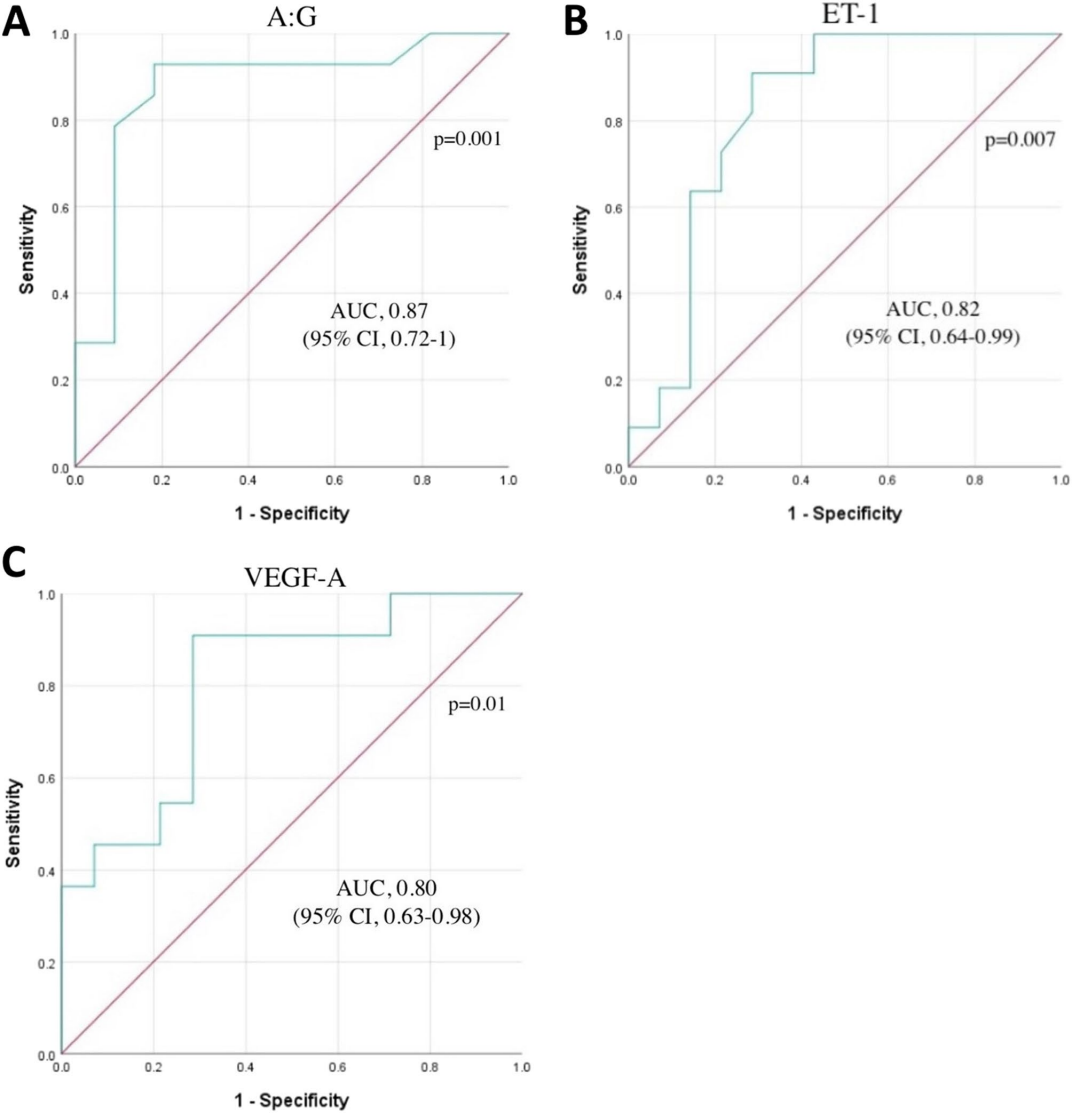
Receiver operating characteristic (ROC) curve analysis to discriminate between surviving and non-surviving cats with hemotropic mycoplasmosis based on serum (**A**) A:G ratio, (**B**) ET-1 and (**C**) VEGF-A concentrations.

The results of the ROC analysis to determine the relationship between some parameters and mortality in nonsurviving cats with hemotropic mycoplasmosis are presented in Fig. 1. The results of the ROC analysis showed that A:G ratio at the cut-off point of 0.46 g/dL, area under the curve (AUC) of 0.877 (95% confidence interval (Cl): 0.723–1.000, *p* = 0.001) with 92% sensitivity and 88% specificity (Fig. 1A); ET-1 at the cut-off point of 89.08 ng/L, area under the curve (AUC) 0.821 (95% Cl: 0.649–0.994, *p* = 0.007) with 90% sensitivity and 72% specificity (Fig. 1B); VEGF-A at the cut-off of 57.44 pg/mL, area under the curve (AUC) 0.805 (95% Cl: 0.630–0.980, *p* = 0.010) with 90% sensitivity and 72% specificity (Fig. 1C) were found to be significant prognostic indicators for mortality prediction in cats with hemotropic mycoplasmosis.

## Discussion

This study evaluated the concentrations of serum syndecan-1, ET-1, ADMA, and VEGF-A, markers of glycocalyx degradation, in cats with feline hemotropic mycoplasmosis. Our results showed that the serum concentrations of syndecan-1 and ET-1 were high in cats with feline hemotropic mycoplasmosis and there were significant correlations with other markers. In addition, the biomarkers ET-1 and VEGF-A were useful in predicting mortality in cats with feline hemotropic mycoplasmosis. Taken together, our results suggest that glycocalyx degradation may contribute significantly to the pathogenesis of feline hemotropic mycoplasmosis.

There is a paucity of information regarding the evaluation of blood gas analysis in cats with feline hemotropic mycoplasmosis. İder et al.^25^ reported that metabolic acidosis and hyperlactatemia developed in cats with hemotropic mycoplasmosis. Studies in anemic patients infected with *Plasmodium* species have reported that systemic hypoxia due to anemia may lead to an increase in anaerobic glycolysis, higher lactate production, and lactic acidosis^26^. It has also been reported that the decreased hepatic clearance of lactic acid in malaria may contribute to the development of lactic acidosis^27^. It has also been reported that *Plasmodium*-infected erythrocytes produce up to 100 times more lactate than uninfected erythrocytes^28^. In the present study, hyperlactatemia and mild metabolic acidosis in cats with feline hemotropic mycoplasmosis may be attributed to a decrease in tissue perfusion due to anemia^25,26^ and an increase in lactate production by infected erythrocytes^26,28^.

Changes in the leukocyte panel in cats with feline hemotropic mycoplasmosis have been reported to be highly variable and of limited diagnostic value^2,29^. On the other hand, it has been reported that infected cats may develop a total leukocyte increase^2,25^, monocytosis, and in some cases lymphocytosis^30^. In the present study, the increase in total leukocyte count, granulocytosis, monocytosis, and lymphocytosis in cats with hemoplasmosis was interpreted as an indicator of inflammation due to infection in consistency with previous studies^2,25^. In addition, macrocytic and hypo/normochromic regenerative anemia due to hemolysis are among the most common findings in cats with hemotropic mycoplasmosis^1,31^. In this study, low RBC, Htc, and Hb levels in cats with hemotropic mycoplasmosis may indicate the development of anemia due to hemolysis. However, although MCV levels did not differ between the study groups, MCV and RDW levels above the reference range in cats with feline hemotropic mycoplasmosis may indicate that the anemia has a regenerative character^1,31^.

Platelet (PLT) levels have been reported to be lower in cats with hemotropic mycoplasmosis than in healthy cats^30^. Although there are no data explaining the development of thrombocytopenia in infected animals, it is accepted that low PLT counts in diseases with hemolysis, such as malaria, may be due to immune complex formation and phagocytosis of platelets by splenic macrophages^32,33^. In the present study, thrombocytopenia in cats with hemotropic mycoplasmosis may be related to phagocytosis of platelets by splenic macrophages^32,33^, similar to the pathogenesis of malaria.

Hyperbilirubinemia is associated with severe hemolytic anemia and liver damage in cats with feline hemotropic mycoplasmosis^1,34^. It has been reported that during severe erythrocyte destruction, the capacity of the liver to metabolize bilirubin is exceeded and hyperbilirubinemia develops^35^. In the present study, the increased total and direct bilirubin concentrations in cats with feline hemotropic mycoplasmosis were interpreted as excessive bilirubin production due to intravascular and extravascular hemolysis and exceeding hepatic bilirubin clearance capacity^35^. In addition, the high AST and LDH enzyme activities determined in cats with hemotropic mycoplasmosis may be related to hemolysis, and the high phosphorus levels may be related to the release of excessive amounts of phosphorus from erythrocytes as a result of intravascular hemolysis^25^.

Albumin, total protein, and A:G ratio show changes associated with inflammatory response in various diseases. A low A:G ratio with hyperglobulinemia and hypoalbuminemia has been reported in cats with feline hemotropic mycoplasmosis compared to healthy cats^25,36^. A low A:G ratio indicates active inflammation in cats and has been described as a significant diagnostic parameter^37^. In addition, low A:G ratio has been reported to be an independent predictor of prognosis in some chronic diseases, cancers, and critically ill patients^38–40^. In the present study, albumin and A:G ratio were significantly lower in cats with feline hemotropic mycoplasmosis compared to healthy cats and were found to be good prognostic indicators for predicting mortality with 92% sensitivity and 88% specificity at a cut-off point of 0.46 ng/mL. Lower serum albumin concentration and A:G ratio in cats with feline hemotropic mycoplasmosis compared to healthy cats can be interpreted as an inflammatory response due to infection and acute phase response^25,37^. It can be concluded that the A:G ratio can be used to predict mortality in cats with feline hemotropic mycoplasmosis.

During the onset of pathological conditions, the eCGx layer is damaged and endothelial glycocalyx components such as heparan sulfate, syndecan, and hyaluronic acid are incorporated into the bloodstream. Recently, there has been increasing interest in studies to monitor and diagnose diseases by measuring the concentrations of biomarkers of glycocalyx damage^7,9,18^. Syndecan-1, which is bound to the endothelial cell surface, has been reported to be targeted by microbial pathogens, particularly in the early stages of infection, and to be involved in the initiation of acute inflammatory responses^41^. The release of syndecan-1 into the circulation has also been reported to reflect more severe eCGx damage and greater functional impairment^8,9^. Syndecan-1 concentrations were found to be elevated in severe malaria cases due to eCGx damage, and this was negatively correlated with RBC and PLT^9^. Researchers have noted that the glycocalyx stabilizer sphingosine-1-phosphate (S-1-P) is decreased in malaria cases due to anemia and/or thrombocytopenia, and this may contribute to glycocalyx deterioration^9^. In this study, significant correlations between high syndecan-1 concentrations and hematological parameters (WBC, RBC, PLT) in cats with hemotropic mycoplasmosis suggest the development of an acute inflammatory response^41^ and eCGx damage^8,9^ in these cats.

Heparanase is another critical factor contributing to endothelial glycocalyx degradation^10^. Endothelin-1, released during glycocalyx degradation, is the primary heparanase activator and keeps it in a vicious cycle to continue glycocalyx degradation^6,7,42^. In addition, ET-1 is thought to mediate endothelial dysfunction both by interfering with the expression and activity of endothelial NO synthase^43^ and by decreasing NO bioavailability through the formation of reactive oxygen species^44^. In this study, the significantly increased serum ET-1 concentrations in cats with feline hemotropic mycoplasmosis compared to the control group and the positive correlations between syndecan-1 and ADMA may reflect the development of glycocalyx damage by heparanase activation and altered NO bioavailability in cats with feline hemotropic mycoplasmosis^6,42,43^.

In human medicine, a significant correlation between ET-1 concentrations and morbidity and mortality has been reported in sepsis^45,46^, and chronic renal failure^47^. On the other hand, in veterinary medicine, ET-1 concentrations were found to be higher in non-surviving premature calves with respiratory distress syndrome than in survivors and were also significant for mortality with 87% sensitivity and 82% specificity^48^. Similarly, ROC analyses showed that ET-1 was a promising prognostic indicator for predicting mortality in cats with feline hemotropic mycoplasmosis with 90% sensitivity and 72% specificity in the present study. The results indicate that ET-1 concentrations can be used as diagnostic and prognostic biomarkers in cats with feline hemotropic mycoplasmosis.

The present study showed a negative correlation between serum ET-1 concentrations and RBC and PLT, and a positive correlation with WBC and lactate. Although the reasons for the relationships between ET-1 concentrations and hematological parameters are not fully elucidated, ET-1 has been shown to play a role in the pathogenesis of hypoxia^48^ and many inflammatory diseases, leading to increased concentrations^45,46,49^. Furthermore, strong correlations between ET-1 and anemia and thrombocytopenia have been demonstrated in patients with chronic renal failure^47^. These findings may reflect the development of an acute inflammatory response in cats with hemotropic mycoplasmosis^45,49^ and a potential link between ET-1, anemia and thrombocytopenia^47^.

Another heparanase activator is asymmetric dimethylarginine (ADMA), an endogenous endothelial NO synthase (eNOS) inhibitor^42^. This biomarker reduces NO production by inhibiting eNOS function^50^. ADMA concentrations were found to decrease and then gradually increase in cases of acute severe malaria and sepsis^50–52^. In the present study, there was no significant difference between ADMA concentrations of healthy and infected cats. The cats with hemoplasmosis included in our study were in the acute clinical stage of the disease and therefore we expected ADMA concentrations to be low^50,52^. The release of free ADMA into the circulation due to erythrocyte lysis during disease^53^ may have contributed to the no statistical difference.

In the present study, ADMA concentrations were positively correlated with serum syndecan-1, ET-1 and VEGF-A concentrations. Two of these biomarkers (ET-1 and VEGF-A) are associated with reduced endothelial NO bioavailability^6,42,54,55^ and syndecan-1 is directly related to glycocalyx degradation^8,9^. Taken together, these findings suggest that in cats with feline hemotropic mycoplasmosis, microvascular reactivity is impaired and endothelial dysfunction develops due to decreased endothelial NO bioavailability.

VEGF-A concentrations have been reported to be elevated in malaria and inflammatory diseases as a result of endothelial dysfunction^13,14^. In a recent study, there was no significant difference in VEGF-A concentrations between children with malaria and healthy ones^12^. Low concentrations have been associated with severe organ damage and mortality in sepsis, but it has not been a good indicator of prognosis^56^. Also, no association was found between low VEGF-A concentrations and disease severity or mortality in severe malaria^54,55^. In these studies^54–56^ low VEGF-A concentrations were associated with decreased NO bioavailability. In the present study, VEGF-A was not a diagnostic marker because VEGF-A concentrations were similar in healthy and infected cats. This may be related to the fact that the levels of PLT, which is the main source of VEGF-A, are low in cats with hemotropic mycoplasmosis^57^. However, VEGF-A concentrations were found to be an important indicator of mortality with a sensitivity of 90% and a specificity of 72%. Surprisingly, serum VEGF-A concentrations were not low in non-surviving infected cats, and those above the cut-off of 57.44 pg/mL died. Therefore, our results do not support previous studies^54–56^. In non-surviving cats, VEGF-A concentrations may be elevated due to protection or repair of endothelial damage^11^. In our study, the deterioration of circulating VEGF-A concentrations and its positive correlation with serum ADMA concentrations may reflect the impaired endothelial NO production and endothelial dysfunction described in severe malaria^55^ and sepsis^56^. In light of these findings, VEGF-A may play a role in the pathogenesis of hematropic mycoplasmosis.

However, the study has several limitations: (1) cats with feline hemotropic mycoplasmosis were not histopathologically examined for glycocalyx damage, (2) the patient population included in the study was small, and (3) heparanase and heparan sulfate concentrations could not be measured in the present study because cat-specific commercial ELISA kits were not available. All of these issues need to be addressed in further studies.

In conclusion, our results suggest that endothelial glycocalyx damage develops in cats with hemotropic mycoplasmosis and that glycocalyx degradation is likely to be an important contributor to disease pathogenesis. Serum syndecan-1 and ET-1 concentrations can be used to diagnose glycocalyx damage. In addition, serum ET-1 and VEGF-A concentrations could be used as prognostic biomarkers in feline hemotropic mycoplasmosis.

## Data Availability

All data generated or analyzed during the current study are included in this article. The data that support the findings of this study are available from the corresponding author, MI, upon reasonable request.
